# Medicinal Food Products; a New Approach from Ordinary Foods to Medicine

**Published:** 2016

**Authors:** Amir M. Mortazavian, Neda Mollakhalili Meybodi

**Affiliations:** a Amir M. Mortazavian; b Neda Mollakhalili Meybodi


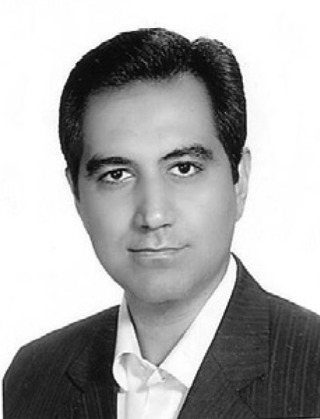


Medicines are designed to cure or prevent distinct symptoms of diseases. However, medication almost is the last solution when prevention or treatments through miscellaneous ways are available. One of the most promising ways is via consumption of healthy food products. From the food technology point of view, foods are consumed for three main reasons: nourishment (providing basic nutritional requirements of body), hedonism (enjoyment during the consumption) and trade (economics of purchasing and sailing). Amongst, the first and the last characteristics can be provided and supported by supplements (as a type of drug products) and drugs, whilst hedonism is solely achievable via consumption of foods. Therefore, the concept of ‘food product’ is inherently meaningless without having acceptable organoleptic (sensory) attributes to consumers’ point of view. This strategy pushed the food technologists to invent new generation of food products called ‘functional food products’; namely those having specific therapeutic or preventive effects, compared with the ordinary similar types, when are consumed regularly in adequate amounts. They can improve wellness in population via improving the basic health and preventing proliferation of diseases. Products with reduced or omitted harmful ingredients (e.g., low-fat, non-fat, low-sodium, low calorie, sugar-less and free cholesterol) as well as those with added healthful chemical and microbial ingredients (e.g., vitamin- enriched, mineral-enriched, phytochemical-enriched, probiotic and prebiotic) are types of functional foods.

The worldwide market of functional foods have been being boosted in such a way that in 2000,2005, and 2010, this market generated US$32.07 billion, US$68.39 billion, and US$155.41 billion, respectively. In 2003, the largest markets for functional foods and supplements announced for United States, Europe (Germany, France, United Kingdom, and the Netherlands were the most important countries within the functional food market), and Japan, accounting for 33.6%, 28.2%, and 20.9% of sales, respectively. The best food matrices to produce functional foods are dairy products, particularly fermented milks. Yogurt products accounted for the largest share of sales among these products, representing about 36%.

Functional food products can be produced as generic foods, namely for mass market and broad range of consumers, or as specific foods, namely for a narrow target group with a specific characteristic or disorder. The latter designated foods are called ‘Medicinal food products’ (it is also so-called as ‘medical food products’). On the other words, they are types of functional foods with specific health claim(s) and higher insurance of medication compared with the ordinary ones. These products must provide effective and fairly fast-emerged curing impacts similar to medicine. The consumers of medicinal food products are mainly patients with limited or reduced ability to metabolize an ordinary foodstuff and those who medically need a certain nutrient. In other words, it has been expected that a distinctive nutritional requirement, resulting from a disease and/or circumstance, provided by medical food consumption.

Formulation and industrially production of medicinal food products are rather difficult; because on one hand, they must maintain their medicinal effects in food matrices (which are much complex than drug matrices from variety, reactivity and perishability stand points) until the end of product shelf life, and on the other hand, they must render sensory characteristics (flavor, texture and appearance) as acceptable and pleasant as the control or ordinary products demanded by the consumers. However, they might possess unpleasant sensory characteristics. Therefore, it is important to compare a medicinal food product with the non-medicinal control through sensory evaluation when developing new food products. Overall, consumers are not interested in consuming a functional food if added ingredients confer disagreeable flavors, even if they result in advantages with respect to their health. In the view of a consumer, sensory characteristics of a food (either plain or functional) are superior to its health considerations; otherwise intake of drug products is more simple and preferable. Moreover, medicinal food products must meet the FDAs regulatory to certify that the product is safe enough to be used for their aimed uses, the claims are exact, not confusing and on the basis of thorough science. It should be pointed out that the labeling reflection of medical foods must be ample to inform the consumers, since these products are accessible for consumers without prescription either on pharmacy shelves or in a food store. These foods are proposed to be consumed under medical administration.

As a conclusion, there is a promising prospect in front of the food technology area for development of medicinal food products, and if is effective, would be more preferable to the consumers’ points of view in comparison with medicines. However, different difficulties could be emerged through formulation, processing and storage regarding medicinal impacts and sensory attributes. Additionally, the price of final product should not be overlooked. Although these products comprise an intrinsic added-value, the price must be reasonable as ‘food’.

*Amir M. Mortazavian is currently working as Associate professor of Department of Food Science and Technology, Faculty of Nutrition Sciences and Technology/National Nutrition and Food Technology Research Institute, Shahid Beheshti University of Medical Sciences, Tehran, Iran. He could be reached at the following e-mail address:* mortazvn@sbmu.ac.ir

